# Supplementary material for: Changes in Protonation States of In-Pathway Residues can Alter Ligand Binding Pathways Obtained From Spontaneous Binding Molecular Dynamics Simulations
Source: Front Mol Biosci. 2022 Jul 4;9:922361. doi: 10.3389/fmolb.2022.922361 (PMC9289141; doi:10.3389/fmolb.2022.922361)
Supplement: Supplementary file 1 [file DataSheet2.PDF]

Supplementary Material

1 Supplementary Figures

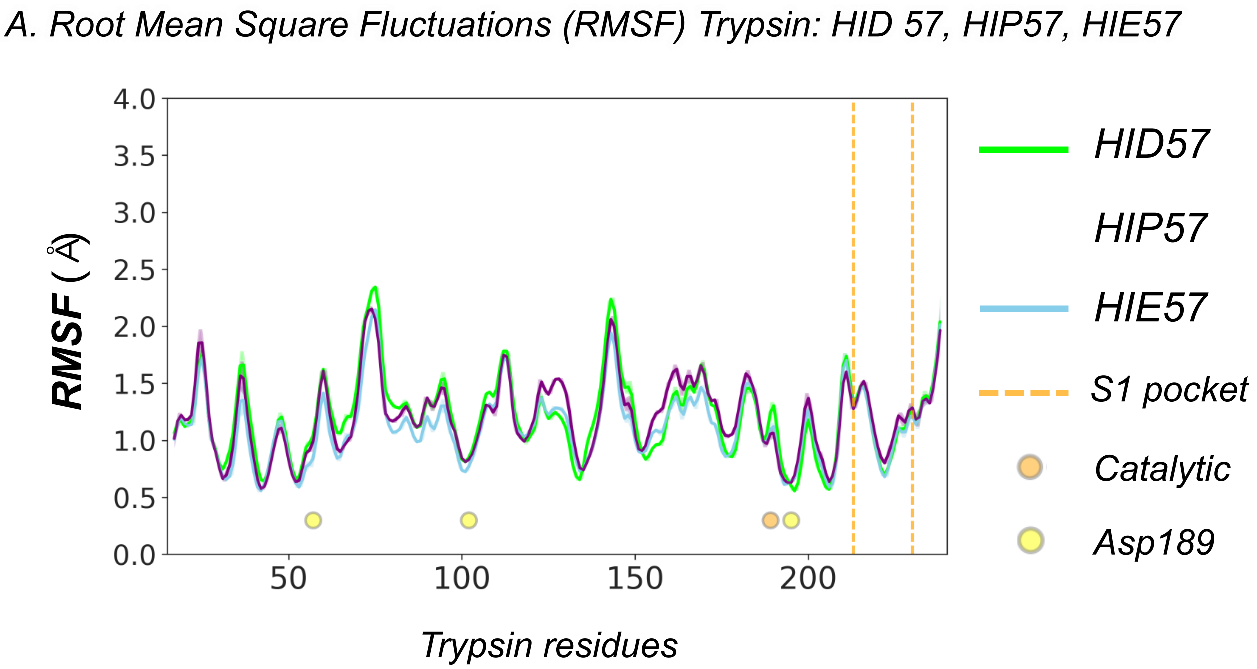

Supplementary Figure 1. Plot of the Root-Mean-Square-Fluctuation (RMSF, in Å) for trypsin HID57 (green), HIDP57 (purple), and HIE57 (blue) obtained from fifty replicas of 200 ns MD simulations. The catalytic residues are highlighted in yellow and Asp189 in orange. Vertical orange dashed lines indicate the position of the S1 pocket loop. In terms of global flexibility, all systems present a similar flexibility.

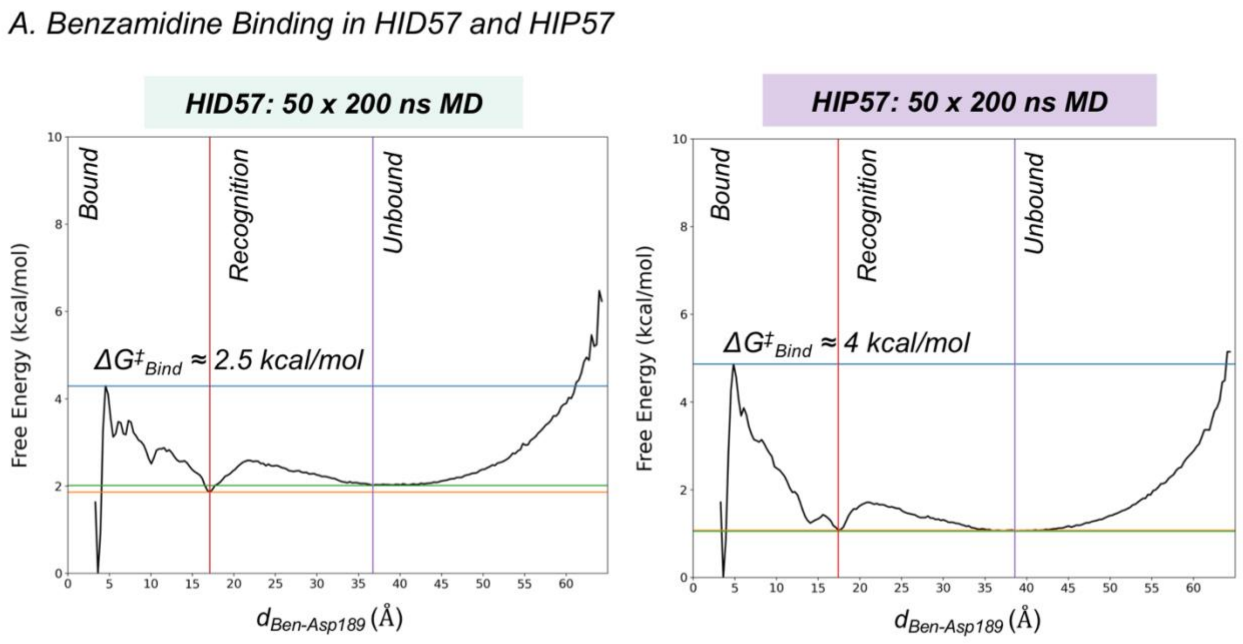

Supplementary Figure 2. (A) Free energy profile reconstructed from 50 replicas of 200 ns of spontaneous binding MD simulations of HID57 and HIP57 using the binding distance ($d_{Ben\text{-}Asp189}$, x axis) between the carbon atom of the amidine group of benzamidine and the carbon of the carboxylate group of Asp189 and the distance between the carbon atom of the amidine group of benzamidine and the epsilon nitrogen of His57 ($d_{Ben\text{-}His57}$, y axis). The estimated free-energy difference ($\Delta G^{\ddagger}_{Bind}$) between the unbound conformation and the transition state that leads to productive binding is provided for each case. The free-energy difference is around 2.5 kcal/mol and 4.0 kcal/mol for HID57 and HIP57, respectively pointing out that the binding of benzamidine is globally slowed down when His57 is positively charged.

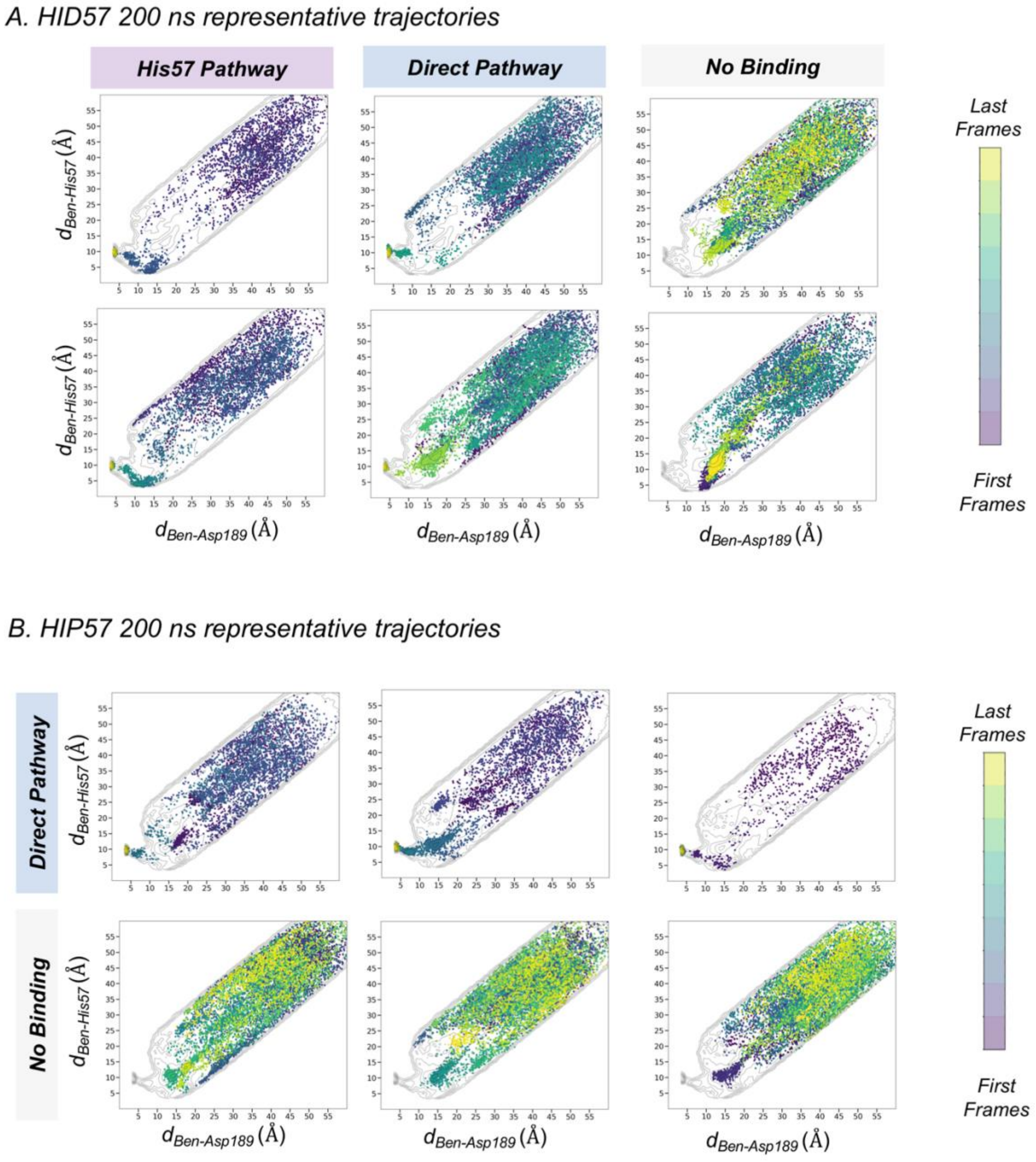

Supplementary Figure 3. Projection of representative spontaneous binding 200 ns MD trajectory on the FEL (see Figure 3 main text) of each HID57 and HIP57. Free energy landscape (FEL) reconstructed from 50 replicas of 200 ns of spontaneous binding MD simulations of HID57 (A) and HIP57 (B) using the binding distance ($d_{Ben\text{-}Asp189}$, x axis) between the carbon atom of the amidine group of benzamidine and the carbon of the carboxylate group of Asp189 and the distance between the carbon atom of the amidine group of benzamidine and the epsilon nitrogen of His57 ($d_{Ben\text{-}His57}$, y axis). Selected examples of the His57 pathway, direct pathway, and replicas where binding is not observed are provide for each protonation state. The time evolution of the ligand binding pathway is represented in a colour scale ranging from purple for the first frames to yellow for the last frames of the MD trajectory.

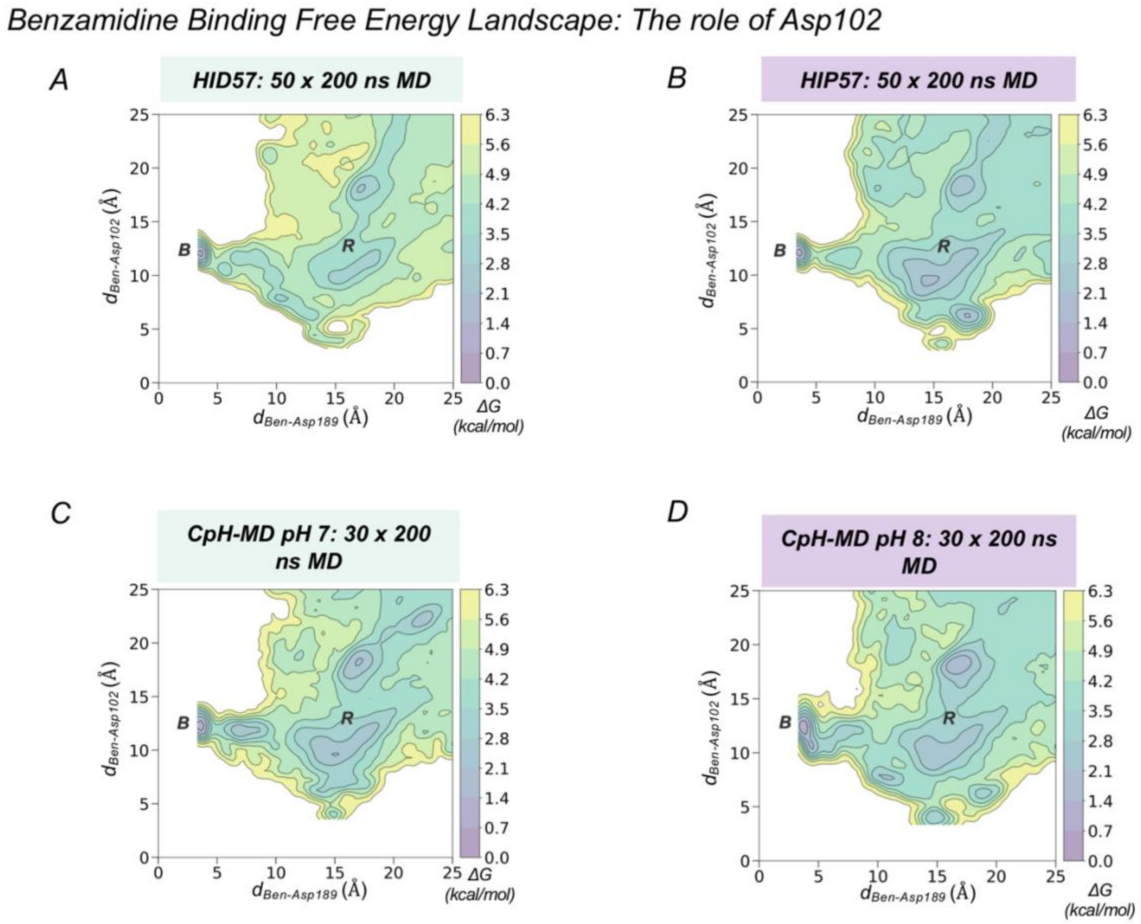

Supplementary Figure 4. (A-B) Free energy landscape (FEL) reconstructed from 50 replicas of 200 ns of spontaneous binding MD simulations of HID57 and HIP57 using the binding distance ($d_{Ben\text{-}Asp189}$, x axis) between the carbon atom of the amidine group of benzamidine and the carbon of the carboxylate group of Asp189 and the distance between the carbon atom of the amidine group of benzamidine and the carbon of the carboxylate group of Asp102 ($d_{Ben\text{-}Asp102}$, y axis). (C-D) Free energy landscape (FEL) reconstructed from 30 replicas of 200 ns of spontaneous binding constant-pH MD simulations at pH 7 and 8 using the binding distance ($d_{Ben\text{-}Asp189}$, x axis) between the carbon atom of the amidine group of benzamidine and the carbon of the carboxylate group of Asp189 and the distance between the carbon atom of the amidine group of benzamidine and the carbon of the carboxylate group of Asp102 ($d_{Ben\text{-}Asp102}$, y axis). Negatively charged Asp102 can play a role in attracting benzamidine in the S3 pocket. From spontaneous binding simulations it can be observed that benzamidine transiently interacts with negatively charged Asp102. However, Asp102 is not always part of benzamidine binding pathway toward the S1 pocket.

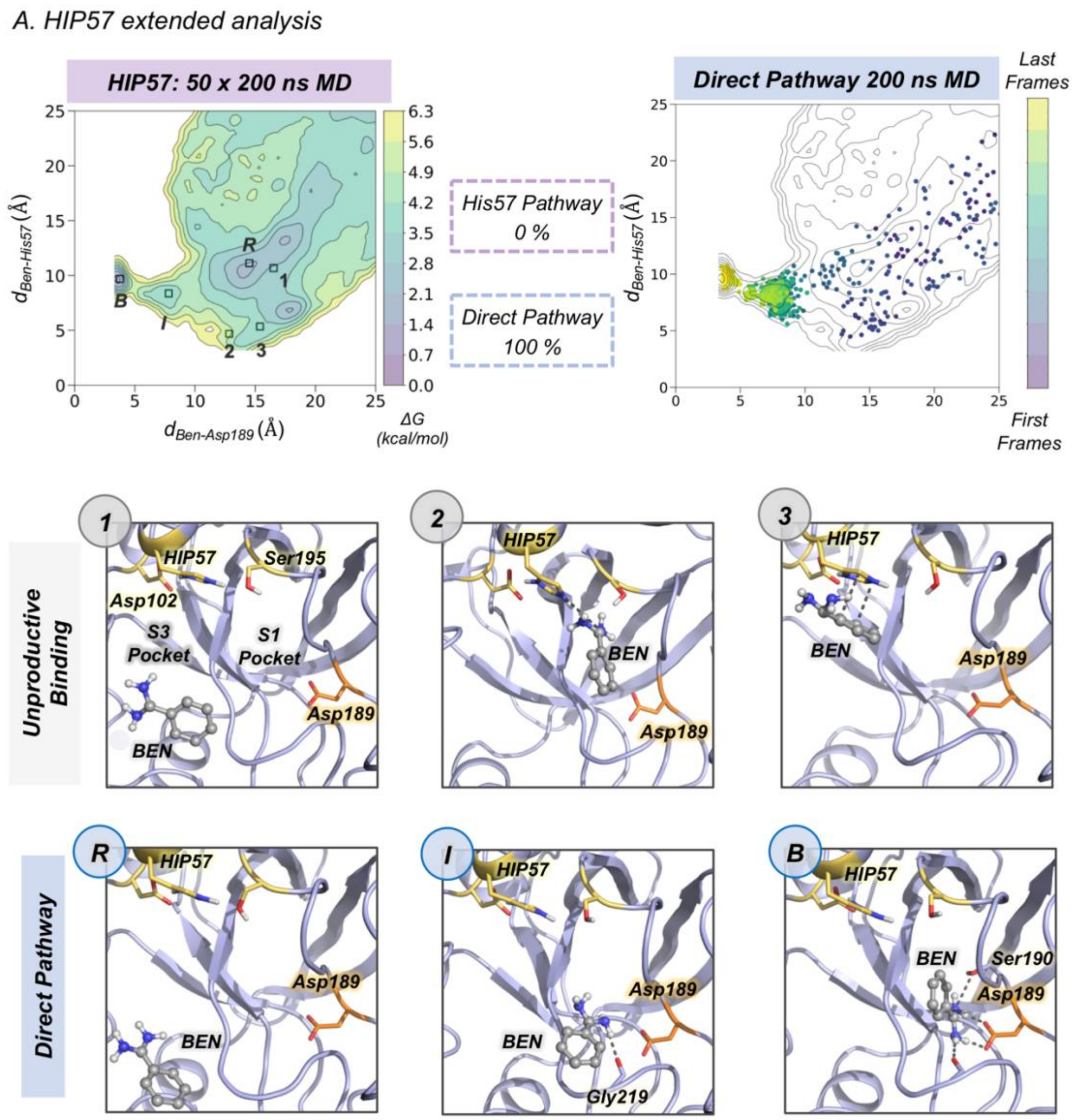

Supplementary Figure 5. (A) Free energy landscape (FEL) reconstructed from 50 replicas of 200 ns of spontaneous binding MD simulations of HIP57 using the binding distance ($d_{Ben-Asp189}$, x axis) between the carbon atom of the amidine group of benzamidine and the carbon of the carboxylate group of Asp189 and the distance between the carbon atom of the amidine group of benzamidine and the epsilon nitrogen of His57 ($d_{Ben-His57}$, y axis). The most relevant states of the FEL are highlighted in black boxes: *U* (unbound), *R* (recognition), *B* (bound) and *I* (intermediate) states while unproductive binding pathways are highlighted in humber. The percentage of binding events (considering only productive binding simulations) that follow each pathway is provided. Projection of a representative spontaneous binding 200 ns MD trajectory on the FEL of HIP57. The time evolution of the ligand binding pathway is represented in a colour scale ranging from purple for the first frames to yellow for the last frames of the MD trajectory. Molecular representation of the most relevant states of the FEL corresponding to the direct pathways. Catalytic residues are shown in yellow, benzamidine in grey, and Asp189 in orange. When benzamidine approaches His57 repulsive interactions between positively charges His57 and benzamidine bring the inhibitor back to the solvent.

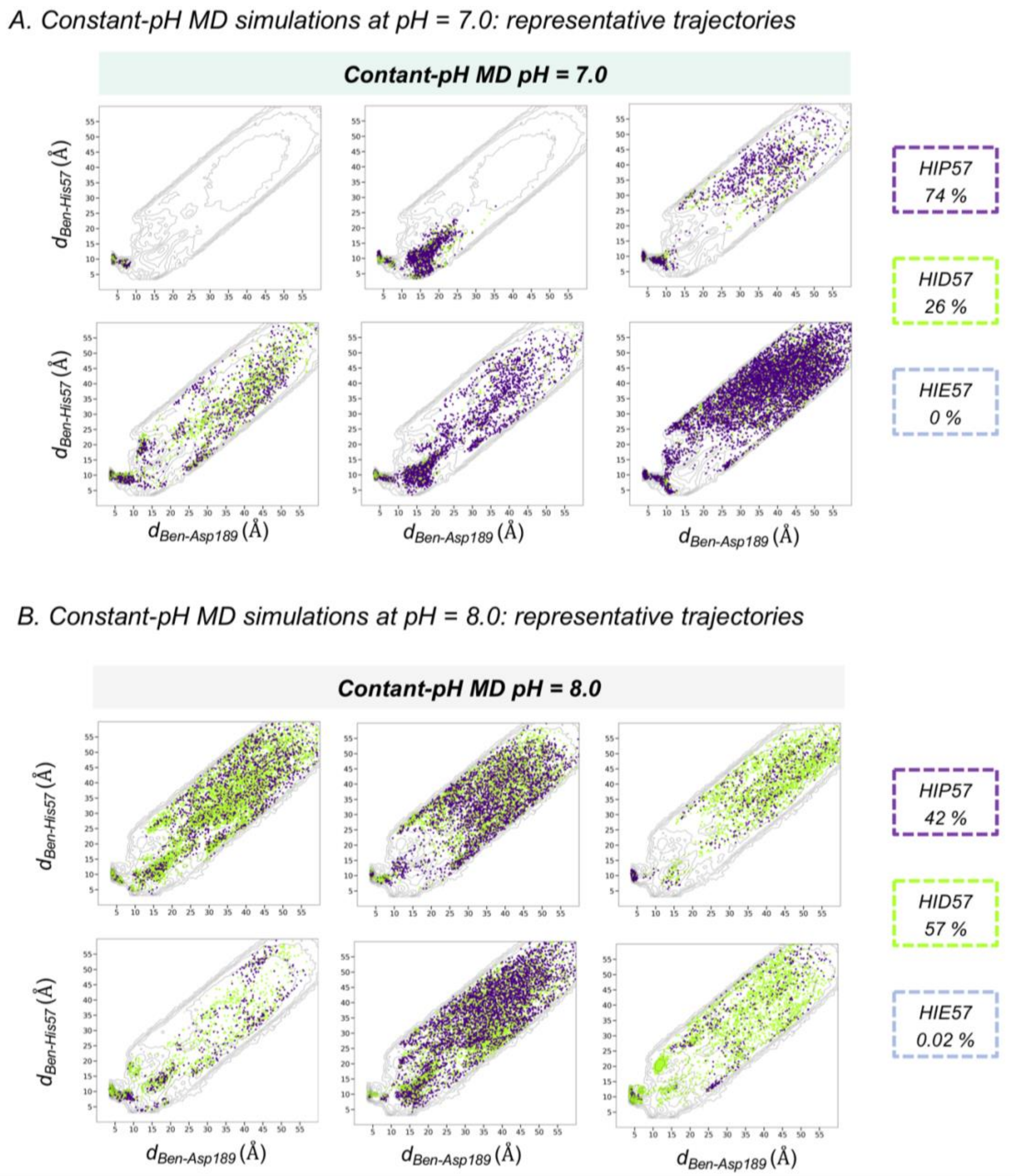

Supplementary Figure 6. Free energy landscape (FEL) reconstructed from 30 replicas of 200 ns of spontaneous binding constant-pH MD simulations at pH = 7.0 (A) and pH = 8.0 (B) using the binding distance ($d_{Ben\text{-}Asp189}$, x axis) between the carbon atom of the amidine group of benzamidine and the carbon of the carboxylate group of Asp189 and the distance between the carbon atom of the amidine group of benzamidine and the epsilon nitrogen of His57 ($d_{Ben\text{-}His57}$, y axis). The most relevant states of the FEL are highlighted in black boxes: *U* (unbound), *R* (recognition), *B* (bound) and *I* (intermediate) states. Projection of a representative 200 ns trajectory of the His57 pathway showing the protonation state of each frame in different colour: HID in green, HIP in purple, and HIE in blue. The equilibrium populations of each protonation state retrieved from the 30 replicas of 200 ns is provided for pH = 7.0 and 8.0.

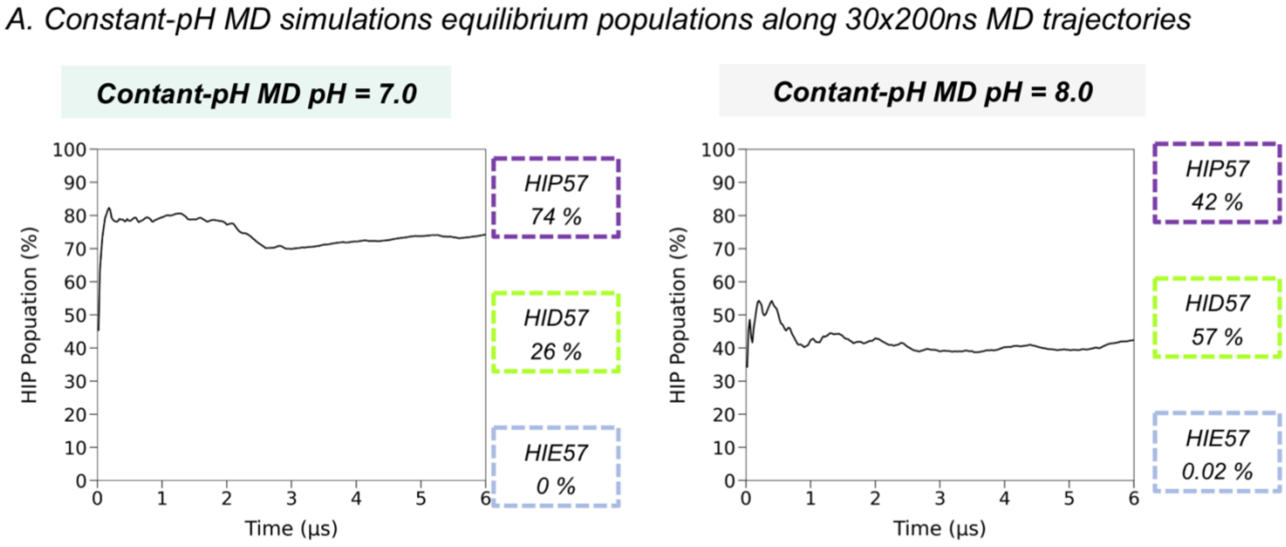

Supplementary Figure 7. Convergence of equilibrium populations of His57 protonation states considering the 30 replicas of 200 ns of MD simulations at both pH 7 and pH 8. These results indicate that the equilibrium populations are converged for both pH 7 and 8. The equilibrium populations of each protonation state retrieved from the 30 replicas of 200 ns is provided for pH = 7.0 and 8.0.

2 Supplementary Methods

Free-energy profile reconstruction. We collectively represented all spontaneous binding MD simulations and constant-pH MD simulations using two coordinates: (1) the binding distance ($d_{Ben\text{-}Asp189}$, x axis) between the amidine carbon of benzamidine and the carboxylate carbon of Asp189; and (2) the distance between the amidine carbon of benzamidine and the epsilon nitrogen of His57, for either HID57 or HIP57 ($d_{Ben\text{-}His57}$, y axis). We selected the epsilon nitrogen because it is directly interacting with the hydroxyl group of catalytic Ser195. The free energy landscape (FEL) is reconstructed from 2D histograms of the abovementioned collective variables by converting the 2D probability distribution to free energy using: $\Delta G\ (x, y) = -[\ln p(x, y) - \ln p_{max}(x, y)]$ where $p(x, y)$ is the probability obtained from the 2D histogram of x and y. To retrieve the free energies in kcal/mol at 300 K, we applied a factor of RT = 0.596. The $\ln p_{max}(x, y)$ is subtracted from the free energy to set the energy minimum to $\Delta G = 0$.
